# Filariasis serosurvey, New Caledonia, South Pacific, 2013

**DOI:** 10.1186/s13071-015-0713-9

**Published:** 2015-02-15

**Authors:** Maguy Daures, Julie Champagnat, Anne Pfannstiel, Frédérique Ringuenoire, Jean-Paul Grangeon, Didier Musso

**Affiliations:** Health Action Service, New Caledonia Health Department, BP N4 - 98851 Nouméa cédex, New Caledonia; Institut Louis Malarde Institut, Tahiti, French Polynesia

**Keywords:** Lymphatic filariasis, Serosurvey, New-Caledonia, Pacific

## Abstract

**Background:**

Lymphatic filariasis (LF) is a major public health problem in the Pacific. As the global prevalence of infection was not known in New Caledonia (NC), a serosurvey study was conducted by determining the prevalence of circulating filarial antigens, as recommended by the World Health Organization.

**Findings:**

A cross sectional study on a 2 degree stratified sample was carried out from June to November 2013. Inclusion criteria were: individuals aged 2 to 80 y/o, who had been hospitalized or sought medical care for a non-infectious cause and who had been living in NC for more than 6 months. LF antigenic detection was performed using the immunocromatographic BinaxNOW filariasis card test (ICT).

Among the 1,035 individuals tested, 7 were antigenic. The overall LF antigenic prevalence was 0.62% (CI 95% [0.60-0.63]).

All patients were unrelated to each other; none of them presented clinical symptoms of LF.

Four of the 7 ICT positive patients reported having travelled to LF endemic areas, 2 patients had never traveled outside NC and the last one had only traveled in non-endemic areas.

For the 7 ICT positive patients, the research of microfilariae in blood smears and filarial DNA by PCR was negative.

**Conclusion:**

The prevalence of filarial antigenemia in NC is less than 1%, the threshold that defines the filarial endemic areas for WHO. Nevertheless, as two patients who had never travelled outside NC and one who had only travelled to non-endemic areas were antigenic, we cannot conclude that NC is totally free of LF.

## Background

Lymphatic Filariasis (LF) is transmitted by mosquitoes and caused by three species of nematodes but only *Wuchereria bancrofti* is found in the Pacific area [1-3]. Filariasis, is common in many parts of the tropics and subtropics, including Pacific island countries and territories (PICTs). This neglected tropical disease is a major cause of disability, social stigmatization and reduced economic life opportunities [4,5]. In 1997, the World Health Organization (WHO) declared LF to be one of six potentially eradicable diseases, and the Global Programme to Eliminate Lymphatic Filariasis (GPELF) was then established with the goal of the “elimination of LF as a public health problem by the year 2020 [6,7]”.

New Caledonia (NC) is a French Territory located in the Southwest Pacific Ocean. It is one of the 22 PICTs comprising 250.000 inhabitants (40.3% Melanesian, 34.6% European, 9.3 mixed-race, 8.7% Wallisian, 2.0% Tahitian, a few Asian and Vanuatuan, and 5.1% indeterminate).

Historically, the levels of filariasis in the Pacific area have been some of the highest in the world [3]. In 1999, the Pacific Programme to Eliminate Lymphatic Filariasis (PacELF) was created [8] and most of the PICTs, but not NC, conducted initial surveys to map the extent of LF.

As elephantiasis was not being diagnosed in NC and *Aedes polynesiensis,* the main vector of LF in the Pacific region was not present [9], it was assumed that the overall prevalence of LF would be low. Nevertheless, since French Polynesia (FP) is still a high endemic area for LF [10] and this ethnic group accounts for 2.0% of the NC population with routine travel exchanges between the two countries, we cannot exclude the possibility that LF is endemic in NC, especially in some remote areas such as Ouvea Island or villages on the northeast coast of New Caledonia, where LF cases have been reported in the past (Canala, Pouebo, Ouega) [11,12].

In order to evaluate NC’s status in regards to LF, we conducted a serosurvey study by determining the prevalence of circulating filarial antigens (CFA) using immunochromatographic card tests (ICT), as recommended by the WHO [13].

## Methods

The serosurvey study was conducted from June to November 2013. A cross sectional study on a 2 degree stratified sample was implemented. Inclusion criteria were: individuals aged 2 to 80 y/o, who had been hospitalized or had sought medical care for a non-infectious cause (temperature below 37.5°C) and who had been living in NC from more than 6 months.

Ethical approval was obtained by the People Protection Committee South-west and Overseas III, Bordeaux, France under reference 13.269. Informed written consent was obtained from all participants for medical interview and blood samples.

The sample size was calculated for a cross sectional study with consideration of the sample plan (design effect) [14,15]. A total of 1,321 individuals were to be included in the study for a seroprevalence precision of +/− 4% (a refusal rate of 10% was planned).

Sampling design: NC was first stratified in 5 areas [Noumea has 39.7% inhabitants of NC, the greater Noumea area (26.9%), East Coast (12.9%), West Coast (13.4%) and Loyalty Islands (7.1%)]. At the first stage, a randomized selection of medical centers and GPs was made. At the second stage, eligible individuals were included in the study by systematic sampling.

Data on socio-demographic characteristics and history of travel were collected using a standardized questionnaire. A blood sample was collected by venous puncture at the end of the interview. For children under the age of 10y/o, antigenic detection was performed only on blood samples collected for other reasons.

After collection, samples were stored and then shipped at −20°C to the Louis Malarde Institute in Tahiti (FP) where they were stored at – 20°C until processing.

LF antigenic detection was performed using the immunocromatographic BinaxNOW filariasis test (Alere North America, Orlando, USA) (Inverness Medical) following the manufacturer’s instruction. In order to avoid false positive reactions the results were recorded 10 minutes after specimen application [16].

After antigenic testing results were obtained, all antigen-positive individuals were contacted and examined by a medical practitioner from the New Caledonia Health Department in order to detect any clinical symptoms of LF. A second blood sample was taken for microscopic and molecular filarial detection. For molecular detection of filarial DNA [17], whole blood was spotted on filter paper and sent to the Louis Malarde Institute in Tahiti FP for individual molecular detection by polymerase chain reaction, as previously described [18].

The data were analyzed using STATA 12.1 software. Data were adjusted by sex, estimates and confidence intervals (CI) were calculated using the “survey” command to consider the sampling plan. The prevalence and 95%CI of filariasis standardized by age was estimated based on the WHO world reference population.

## Findings

A total of 1035 individuals were included in the study. Melanesians were over-represented (48.3%) and Europeans underrepresented (22.9%) in the sample compared to the general population (p < 0.0001).

**Figure 1:**
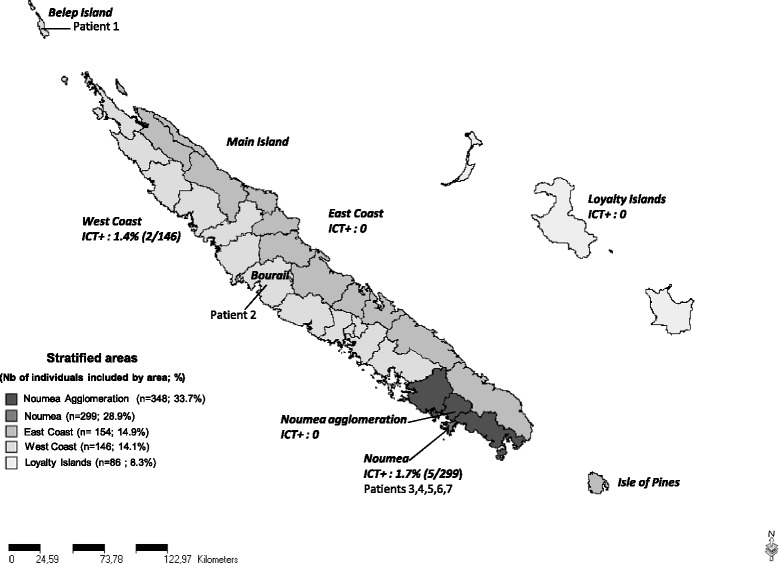
**Distribution of individuals included, prevalence of antigenemia in the different NC areas and location of the ICT positive patients.**

The overall LF antigenic prevalence (after considering the sample plan) was 0.62% (CI 95% [0.60-0.63]). The ICT filariasis test was positive for 7 patients. Prevalence of antigenemia in the different NC areas and locations of the ICT positive patients is shown in Figure 1.

For the 7 ICT positive, all the data recorded through the medical questionnaire and their laboratory data are given in Table 1. The median age of those patients was 35 y/o (ranging from 28 to 64 y/o), the sex ratio (F/M) was 1.33, 5 lived in Noumea, 1 in Belep Islands and 1 in a village on the West Coast. Two were Melanesians and five were Europeans, none of them were from the Tahitian population living in NC. All patients were unrelated to each other; none of them showed any clinical symptoms of LF.

**Table 1:** **Clinical and laboratory findings for the 7 antigenemic patients**

**Patients**	**1**	**2**	**3**	**4**	**5**	**6**	**7**
age	64	62	34	28	35	31	47
sex	M	F	M	F	F	M	F
Community	Melanesian	European	Metis	European	Melanesian	Metis	European
Birthplace	NC	NC	NC	NC	NC	NC	France
NC arrival date							1991
Laboratory results							
ICT	Positive	Positive	Positive	Positive	Positive	Positive	Positive
Microfilaremia	Negative	Negative	Negative	Negative	Negative	Negative	Negative
LF PCR	Negative	Negative	Negative	Negative	Negative	Negative	Negative
Lived/worked in LF area known in NC*	Yes	Yes	Yes	No	Yes	No	Yes
Travels in LF endemic countries							
Travel 1		Malaisia	Vanuatu			Vanuatu	Vanuatu (x3)
Year		1988	2011			1994	1994, 1998, 2010
Travel 2		Vanuatu (x3)				Indonesia	French Polynesia (x10)
Year		2004, 2005, 2007				1996	1992 - > 2011
Travel 3						Viet-Nam	Sri Lanka
Year						2008	1990

*Ouvea Island, Koumac, Canala, Ouegoa, Pouebo. Never left the territory. Visited only non-endemic countries.

Four of the 7 ICT positive patients reported having travelled in the past in an LF endemic country (Vanuatu, FP, Indonesia, Viet Nam, Malaysia and Sri Lanka), 2 had never traveled outside NC to an LF endemic country [19].

For the 7 ICT positive patients tested for live microfilariae and filarial DNA were negative.

Each ICT positive patient received treatment consisting of a single dose of albendazole (400 mg) and diethyl carbamazine (400 mg).

## Discussion

According to WHO, non-endemic areas for LF are those areas where surveys have shown an infection rate of less than 1% [13]. The overall LF prevalence estimated in NC was 0.62% (CI 95% [0.60-0.63]), which allows us to classify NC as a non-endemic PICTs for LF, according to WHO criteria.

Out of the 1,035 patients included in this study, only 7 tested positive for LF antigenemia.

Patients 2, 3, 6 and 7 reported having travelled in the past to LF endemic PICTs or countries (principally Vanuatu and FP) where they may have been contaminated. Vanuatu was one of the first PICTs to initiate the GPELF; after two MDA campaigns LF antigenic prevalence in sentinel sites was 8% in 2002 [20]. FP is another PICTs that has a high LF endemic area. Despite eight rounds of MDA from 2000 to 2007, the overall prevalence was 11.3% in 2008 [10]. The main drawback of LF antigenic detection is that it can remain positive years after the infection because adult LF worms, once dead, release substances detected by the CFA test [21].

Patients 1 and 5 had never left NC and patient 4 had only travelled in non-endemic areas. For them it is not possible to say whether or not they were infected in NC or if the positive result of the antigenemia was a false positive. The occurrence of false positive results in our study is to be considered because the predictive positive value of a test decreases when the prevalence of the disease in the studied population is low.

Patient 5 was born and had lived 29 years on Ouvea Island and may have been contaminated during those years. A study conducted in 1996 in this island showed that 3.7% of the 382 adults tested were microfilaremic [11].

Patients 1,2,3,5,7 had worked or lived in the northern part of NC (Koumac, Ouegoa, Pouebo) or on East Coast (Canala), where filariasis cases have been reported according to a study conducted in 1979–1980 [12].

The seven antigenemic patients were asymptomatic, tested negative for microfilairemia and filarial DNA, and they received preventive treatment, then, no follow protocol has been implemented.

## Conclusion

In view of the results, we conclude that the LF prevalence in NC is less than 1%, so we assume that LF is not a public health concern in NC and that there is no need to set up the GPELF. Nevertheless, as the overall population has not been tested and as three patients had never been exposed in endemic areas, we cannot conclude that NC is totally free of LF, especially in some very remotes areas. To our knowledge, *Aedes vigilax* is the potential vector of LF in NC [12], *Ae. polynesiensis*, the main vector of LF in the Pacific is not present in NC, suggesting that the potential for filariasis emergence is low in this country.

## Abbreviations

CFA: Circulating filarial antigens
CI: Confidence interval
DNA: Deoxyribonucleic acid
FP: French polynesia
GPs: General practitioners
GPELF: Global Programme to Eliminate Lymphatic Filariasis
ICT: Immunochromatographic test
LF: Lymphatic filariasis
MDA: Mass Drug Administration
NC: New Caledonia
PacELF: Pacific Programme to Eliminate Lymphatic Filariasis
PICTs: Pacific Island Countries and Territories
PCR: Polymerase chain reaction
WHO: World Health Organization.
